# H_2_S-releasing nanoemulsions: a new formulation to inhibit tumor cells proliferation and improve tissue repair

**DOI:** 10.18632/oncotarget.12609

**Published:** 2016-10-12

**Authors:** Matteo Ciocci, Egidio Iorio, Felicia Carotenuto, Haneen A. Khashoggi, Francesca Nanni, Sonia Melino

**Affiliations:** ^1^ Department of Chemical Sciences and Technologies, University of Rome Tor Vergata, Rome, Italy; ^2^ Department of Cell Biology and Neurosciences, Istituto Superiore di Sanità, Rome, Italy; ^3^ Department of Clinical Sciences and Translational Medicine, University of Rome Tor Vergata, Rome, Italy; ^4^ Department of Industrial Engineering, University of Rome Tor Vergata, Rome, Italy

**Keywords:** hydrogen sulfide, garlic, omega-3 fatty acid, antioxidants, cancer

## Abstract

The improvement of solubility and/or dissolution rate of poorly soluble natural compounds is an ideal strategy to make them optimal candidates as new potential drugs. Accordingly, the allyl sulfur compounds and omega-3 fatty acids are natural hydrophobic compounds that exhibit two important combined properties: cardiovascular protection and antitumor activity. Here, we have synthesized and characterized a novel formulation of diallyl disulfide (DADS) and α-linolenic acid (ALA) as protein-nanoemulsions (BAD-NEs), using ultrasounds. BAD-NEs are stable over time at room temperature and show antioxidant and radical scavenging property. These NEs are also optimal H_2_S slow-release donors and show a significant anti-proliferative effect on different human cancer cell lines: MCF-7 breast cancer and HuT 78 T-cell lymphoma cells. BAD-NEs are able to regulate the ERK1/2 pathway, inducing apoptosis and cell cycle arrest at the G_0_/G_1_ phase. We have also investigated their effect on cell proliferation of human adult stem/progenitor cells. Interestingly, BAD-NEs are able to improve the Lin^–^ Sca1^+^ human cardiac progenitor cells (hCPC) proliferation. This stem cell growth stimulation is combined with the expression and activation of proteins involved in tissue-repair, such as P-AKT, α-sma and connexin 43. Altogether, our results suggest that these antioxidant nanoemulsions might have potential application in selective cancer therapy and for promoting the muscle tissue repair.

## INTRODUCTION

Epidemiological and preclinical studies support the effect of garlic (*Allium sativum* L.) as a cardiovascular-protective agent with chemopreventive and anticarcinogenic effects. Indeed, allyl sulfur compounds are potentially important for the prevention of both tumour and cardio-vascular diseases [1–16]. In keeping, fresh garlic extracts and oil are reported to inhibit the growth of MCF-7 breast cancer, hepatoma HepG2, colon-carcinoma Caco-2 and pro-myelocytic leukaemia HL-60 cells [17–20]. Allyl sulfur compounds show anti-proliferative effects on cancer cells by blocking the G_1_/S or G_2_/M cell cycle phases [11, 20, 21–24]. The garlic Organo-Sulfur Compounds (OSCs) seems also able to affect chromosomal stability, resulting in deregulated chromosomal organization and block at metaphase [25]. Among OSCs derived from garlic, diallyl-disulfide (DADS) seems to be the most effective at reducing the growth of human tumor cells derived from skin, colon and lung [12, 26]. DADS inhibits the *in vitro* growth of colon, lung, oesophageal, gastric, and leukemia cancer cell lines [27–32], as well as both estrogen receptor (ER)-positive and –negative human breast cancer lines [33]. Additional work suggests that the G_2_/M phase arrest induced by DADS suppresses p34cdc2 kinase activity [34] and increases cyclin B1 protein expression in cultured HCT-15 cells [2]. Omega-3 fatty acids as well as garlic OSCs are natural compounds that exhibit two combined properties: cardiovascular protection [35, 36] and antitumor activity [37, 38]. Acyl chain length and unsaturation of the n-3 poly-unsaturated fatty acids (PUFA) are relevant factors for the activation of different molecular mechanisms that lead to decrease of cancer cell proliferation by changes of lipid raft biochemical and biophysical features [39]. Alpha linolenic acid (ALA) (18:3 Δ9, 12, 15) is one of the main n-3 bioactive long chain-PUFA in food sources that, as well as the other omega-3 fatty acids, enhances cardiovascular health either enhancing the endothelial function or reducing restenosis, coronary disease-associated mortality and the risk of heart attacks [40, 41]. Finally, other studies confirm ALA as a potential dietary agent of chemoprevention on human breast cancer cells. In particular, its anti-carcinogenic property seems related to its ability to affect the growth of breast and colon cancers [36–38, 41] as well as to affect cell death, with cytochrome c translocation, caspase-3 activation, and PARP degradation [42].

Although the above properties of DADS and ALA make them two potential natural drugs for clinical application, their low aqueous solubility and stability complicates their *in vivo* pharmacokinetics, pharmacodynamics and bio-distribution. Indeed, bioavailability of poorly water-soluble drugs is a crucial problem in pharmaceutical formulations. The most frequent causes of low oral bioavailability, in fact, are attributed to poor solubility and low permeability. The aqueous solubility is a major indicator for the solubility in the intestinal fluids and, consequently, for its bioavailability. Thus, an important goal for making new candidate drugs is to improve the solubility and/or dissolution rate for poorly soluble natural compounds. The most common approaches to achieve their enhanced oral bioavailability include the use of micronization, nanosizing, crystal engineering, solid dispersions, cyclodextrins, solid lipid nanoparticles and other colloidal drug delivery systems such as micro-and nano-emulsions, self microemulsifying drug delivery systems and liposomes [43, 44]. The nanoemulsion (NE) leads to a heterogeneous and thermodynamically-stable oil-in-water mixture in which the average oil droplet diameter is in the low-nanometer (< 0.2 μm) range [44, 45]. Nanoemulsions represent hopeful drug-delivery platforms, facilitating the solubilization, encapsulation and delivery of lipophilic cargo molecules to target cells while enhancing *in vivo* cargo bioavailability [45].

In this present manuscript we report the synthesis of a novel protein/ALA/DADS-in-water emulsion. BSA/ALA/DADS nanoemulsion, BAD-NE, has been produced and characterized using RP-HPLC, fluorescence and Scan Electro Microscopy (SEM), and NMR spectroscopy. Our new formulation exhibits antioxidant properties and is able to release the *gasotrasmitter* hydrogen sulfide. The anti-tumor properties of this novel formulation have been evaluated on human MCF-7 breast cancer and HuT 78 T-cell lymphoma cell lines, demonstrating its ability to induce cell cycle arrest and cell death by ERK1/2 pathway and caspase-3 activation. Finally, a critical requirement for the anticancer agents is the selectivity against cancer, rather than normal cells. Indeed, several reports suggest that garlic derived OSCs, such as DADS and DATS, are more active against cancer cells (e.g. ESCC, A375, DU145, PC-3, MCF-7, MDA-MB-231 H358, H460 cancer cell lines) than their normal counterparts [9, 46–52]. Additional evidence comes from the selectivity of ajoene for normal peripheral blood mononuclear cells (PBMC) from healthy humans compared to leukemic cells collected from patients [53]. Several studies have also demonstrated the beneficial effects of ALA on the endothelial and striates muscle cells [54, 55]. On these bases, the effects of NE on human adult progenitor cells have been here investigated demonstrating the absence of cytotoxic effect on Lin^-^ Sca1^+^ cardiac progenitor cells (hCPC). In contrast to the treatment of cancer cells, at the same concentrations, BAD-NE is able to increase the proliferation and stimulate the expression of proteins involved in the initial process of the differentiation, thus suggesting a potential and selective therapeutic application for antitumor therapy and tissue repair.

## RESULTS AND DISCUSSION

### Synthesis and characterization of Protein-NEs with OSCs and Omega-3 fatty acids

**Figure 1:**
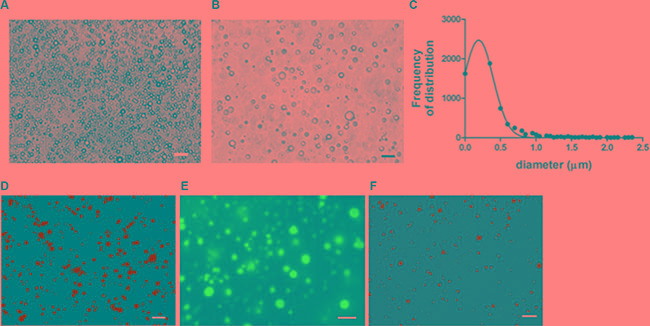
Morphological characterization of NEs Optical micrographs of (**A**) BAD- and (**B**) BD-NEs; (**C**) size distribution of BAD-NEs; fluorescence micrographs of BAD-NEs stained with (**D**) nile red, (**E**) prodan and (**F**) FITC. Obtained at 100× of magnification, scale bars = 10 μm.

**Figure 2:**
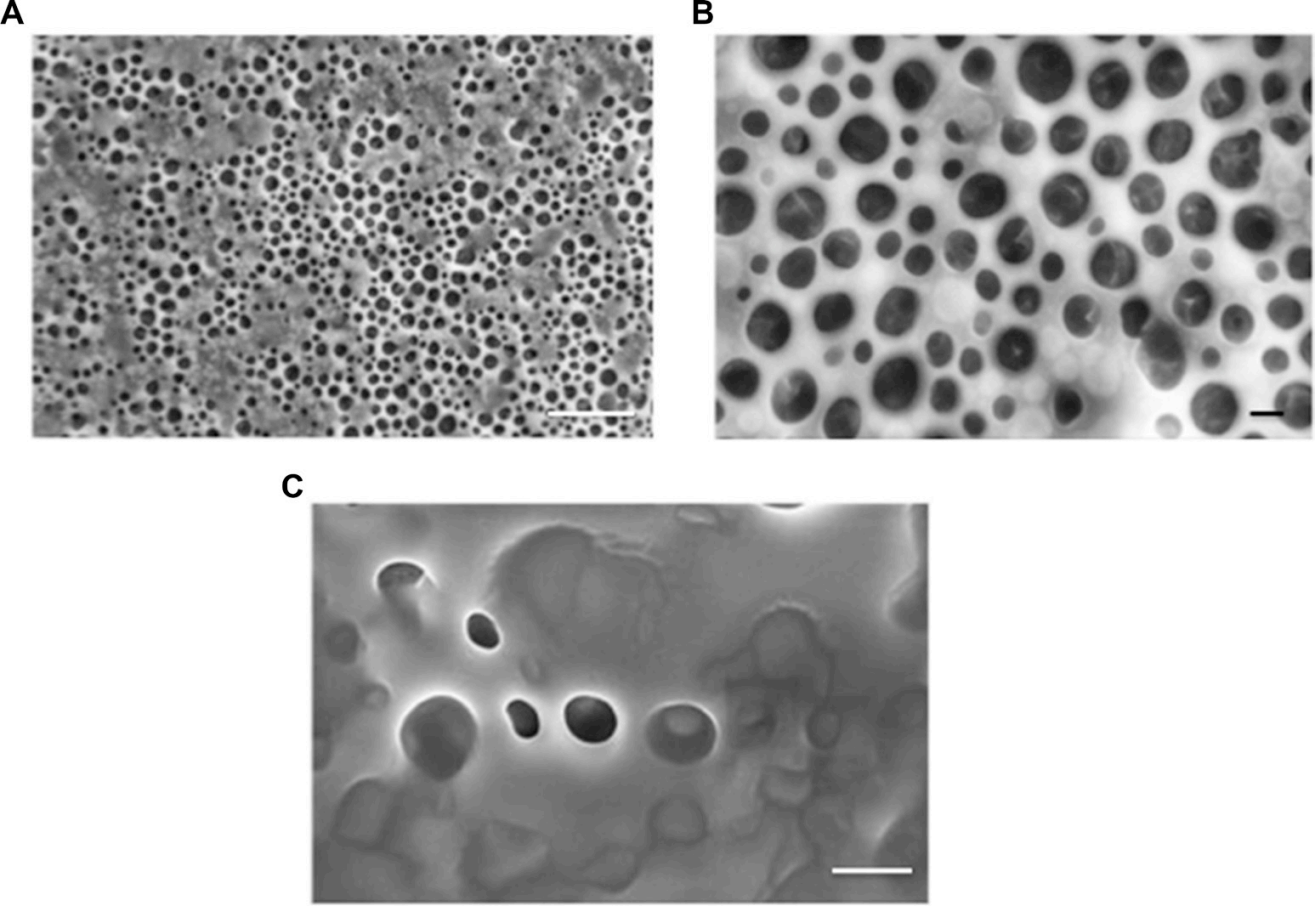
Ultrastructural properties of NEs Representative HR-SEM micrographs of BAD-NEs captured at 10 kV (**A**) and (**B**) and at 5 kV (**C**). Scale bars are: 10 μm in (A) and 1 μm in (B) and (C).

**Figure 3:**
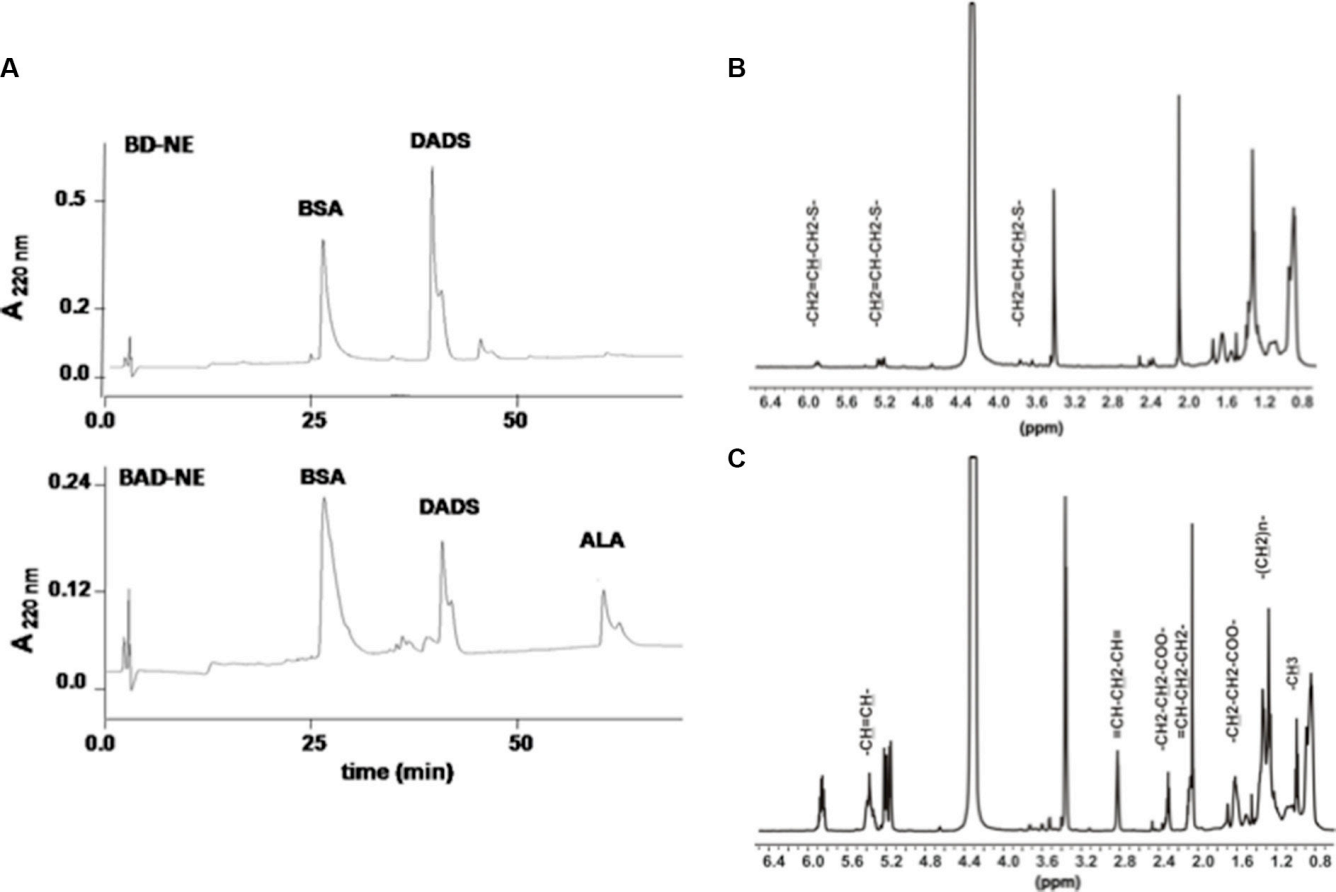
Chemical characterization of NEs RP-HPLC chromatograms of (**A**) BD-NEs and BAD-NEs after solubilization in 40% CH_3_CN and 0.05% TFA v/v, obtained using C_18_ column at 0.8 ml/min flow rate and the following gradient: 0–5 min, 0%; 5–25 min, 60%; 25–55 min, 90% and 55–75 min 90% of solv. (**B)** (80% of CH_3_CN and 0.1% of TFA v/v). The peaks are identified comparing the retention times of the single compounds obtained by RP-HPLC analysis performed at the same conditions.^1^H NMR spectra of (B) BD-NEs and (**C**) BAD-NEs in CDCl_3_/CD_3_OD mixture performed at 25°C.

Protein-DADS-NEs, with and without ALA, have been prepared by sonication of a 5% w/v bovine serum albumin (BSA) solution. The protein-NEs, hereinafter BAD (BSA-ALA-DADS)- and BD (BSA-DADS)-NEs, have been characterized by fluorescence microscopy, RP-HPLC and ^1^H-NMR spectroscopy. Figure 1A and 1B show the optical micrographs of BAD- and BD-NEs, while the size distribution of BAD-NEs is reported in Figure 1C, showing a mean diameter for the BAD-NEs spheres of 0.26 ± 0.06 μm. The presence of the hydrophobic component of BAD-NEs has been assessed by the addition of Nile Red or Prodan fluorophores before the sonication of the mixture (Figures 1D and 1E). The protein on the NEs shell has been detected by fluorochrome labeling of the NEs using fluorescein isothiocyanate (FITC) followed by washing (Figure 1F). Moreover, SEM analysis of BAD-NE confirms the presence of spheres with a diameter less than 1 μm (Figure 2) although, SEM sample preparation by drying and under vacuum metallization lead to an increase in size of the spheres. The presence of both DADS and ALA and their relative concentrations in the NEs has been assessed by RP-HPLC analysis using specific calibration curves for DADS and ALA (Supplementary Figure S1). Figure 3A shows the chromatograms of BD- and BAD-NEs after solubilization in 40% CH_3_CN v/v with 0.05% TFA v/v. Furthermore, the NEs ^1^H-NMR spectra of BD- and BAD-NEs, performed in MeOD/CDCl_3_ mixture, (Figure 3B and 3C) demonstrate the presence of peaks with chemical shifts that are characteristic of the side chains of ALA and DADS. In particular, the spectra show both saturated (1.29 ppm) and polyunsaturated (olefinic, allylic and bis-allylic) resonances of chemical groups of ALA in BAD-NE (Figure 3C and Table 1). The analysis of BAD-NE NMR spectra also indicates the presence of peaks belonging to DADS, also present in ^1^H-NMR spectrum of BD-NE (Figure 3B). Table 1 reports the chemical shifts of the observed resonances that are indicative of the chemical structure of the molecules present in the NEs. The presence of individual resonances of ALA and DADS molecules in the NMR spectra indicates that the NE synthesis process not alter the structures of these molecules.

**Table 1:** ^1^H NMR chemical shifts of ALA and DADS in BAD-NEs solubilized in CDCl_3_/CD_3_OD

Compounds	groups	^1^H chemical shift (ppm)^a^	Multiplicity^a^
ALA			
	-CH_3_	0.98	t
	-(CH_2_)_n_-	1.30	m
	-CH_2_-CH_2_-COO^-^	1.61	q
	=CH-CH_2_-CH_2_-	2.04	m
	-CH_2_-CH_2_-COO^-^	2.35	t
	=CH -CH_2_-CH=	2.80	M
	-CH=CH-	5.36	m
DADS			
	-CH_2_=CH-CH_2_-S	3.76	d
	-CH_2_=CH-CH_2_-S	6.02	m
	-CH_2_=CH-CH_2_-S	5.24	dd

a Proton chemical shift are reported with reference to TMS at 0 ppm and multiplicity definitions are: s, singlet; q, quintet; t, triplet; m, other multiplets. The multiplicity given here was observed in conventional one-dimensional spectra recorded at 700 MHz.

### NEs as potential H_2_S-releasing donors

**Figure 4:**
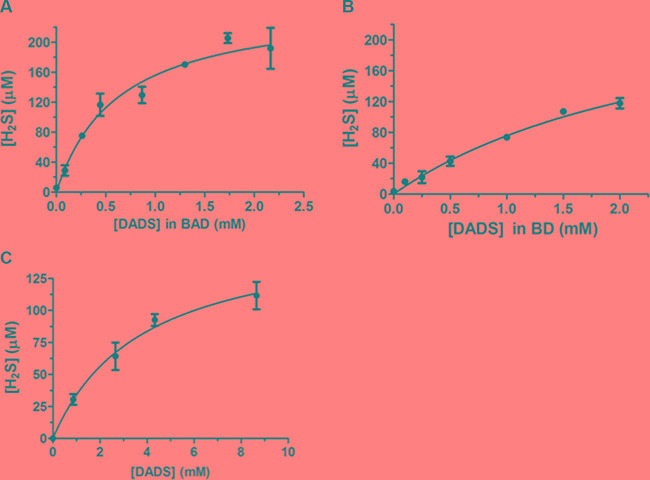
H_2_S slow-releasing by NEs H_2_S-release detected by methylene blue assay at different concentrations of (**A**) BAD-NEs, (**B**) BD-NEs and (**C**) DADS in DMSO, in the presence of 1 mM DTT in 50 mM Tris-HCl, pH 7.4 buffer. The H_2_S concentrations are calculated using a calibration curve obtained at different concentrations of Na_2_S (see also Supplementary Figure S2). Each bar represents the ± SD of three experiments.

Several studies suggest that garlic OSCs and their conjugates are optimal H_2_S slow-releasing agents [20, 56, 57]. Taking into account these studies, we have assessed the H_2_S-release from BAD- and BD-NEs (Figure 4). At the same concentrations of DADS, calculated by using RP-HPLC analysis, BAD-NE is able to produce more H_2_S than both BD-NE and DADS solubilized in DMSO (Figure 4). These results can be explained by the presence of stable sulfane sulfur species on the protein shell of the emulsions that can increase the H_2_S-release, and by the antioxidant property of ALA, which is present in the BAD-NEs. Hence, our results suggest that BAD-NEs could represent an excellent H_2_S-releasing preparation.

The endogenous *gasotransmitter* H_2_S is produced within mammalian cells and, being a signaling molecule, plays a relevant role in several biological processes [56, 58]. A relevant therapeutic potential has been suggested for exogenous sources of H_2_S in neurodegenerative pathologies [58–60], such as Parkinson's and Alzheimer's disease, cardiovascular [61–63] and gastrointestinal [64, 65] diseases. At the molecular level, H_2_S can affect both cell cycle and cell death, with pro- [66–68] and anti- [69, 70] apoptotic activity [71] depending on the cell type, the experimental conditions and, particularly, on the H_2_S concentration used. Thus, for the peculiar properties of this *gasotransmitter* and its pharmaco-dynamic feature, exogenous H_2_S-donors, such as BAD-NE, could disclose attractive pharmacological perspectives.

### Antioxidant properties of BAD-NEs

**Figure 5:**
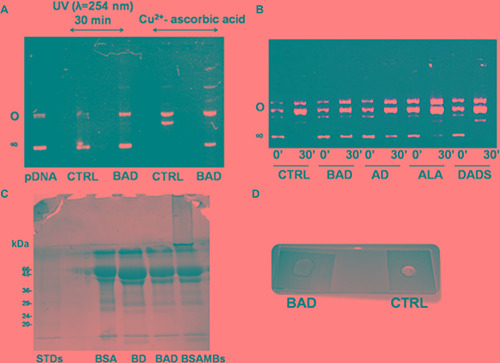
Antioxidant properties of BAD-NEs (**A**) Inhibition of the pDNA cleavage by BAD-NEs, 0.5 μg of pDNA in buffer 50 mM Tris HCl buffer, pH 7.4, after 30 min of UV irradiation at 254 nm (lane 1), in the absence (lane 2) and in the presence of 1 μl of BAD-NE (30 mM DADS and 32 mM ALA) (lane 3); 0.5 μg pDNA after 30 min of incubation with 100 μM CuCl_2_ and 10 mM ascorbic acid in 50 mM Tris HCl, pH 7.4, buffer at 37°C in the absence (lane 4) and in the presence (lane 5) of 1 μl of BAD-NE. (**B**) 0.5 μg of pDNA after addition of 100 μM CuCl_2_ and 10 mM ascorbic acid in 50 mM Tris HCl, pH 7.4, buffer after 0 (lane 1) and 30 min (lane 2) of incubation at 37°C alone and in the presence of BAD-NE (lanes 3 and 4) or ALA-DADS (AD) mixture (lanes 5 and 6), ALA (A) (lanes 7 and 8) and DADS (lanes 9 and 10). The ALA and DADS concentrations are the same in all samples; (**C**) SDS-PAGE of 25 μl of BD-NE (lane 2) BAD-NE (lane 3) and MBs (lane 4) obtained using 5% w/v of BSA solution; (**D**) inhibition of the free-radical polymerization of PEG-fibrinogen hydrogel after addition of 0.1% w/v of Irgacure^®^ 2959 photo-initiator and 5 min of UV (365 nm, 5 mW/cm^2^) exposure in the presence (BAD) or in the absence (CTRL) of 8% v/v BAD-NE.

The antioxidant property of the H_2_S-releasing BAD-NE as scavenger of ROS has been here investigated using three complementary experimental approaches: i) plasmid DNA (pDNA) protection from radicals produced by both UV irradiation and redox-cycling of copper ions in the presence of ascorbic acid; ii) inhibition of the oxidative formation of protein cross-links; and iii) inhibition of the polymerization of polysaccharides induced by radicals (Figure 5). The ability of BAD-NEs to inhibit single- and double-strand breaks in pDNA has been assessed by either UV-exposure or copper redox cycling activity using Cu^2+^ ions and ascorbic acid, in the presence or absence of BAD-NEs. The pro-oxidant activity of Cu^2+^ is due to the formation of Cu^1+^ by reduction from ascorbate in one-electron reaction accompanied by the formation of ascorbate oxidation intermediate, ascorbate radical. A single-strand break causes the conversion of supercoiled pDNA to the open circular form, whereas a double-stranded break causes conversion of supercoiled to the linear form of pDNA. These three forms run at different rates on agarose gels. The untreated plasmid has been included as a control, resulting predominately in a single band of pDNA supercoiled form on the gels (Figure 5 Lane 1). The incubation of the supercoiled pDNA with Cu^2+^ ions and ascorbic acid or under UV (at wavelength of 254 nm) is completely converted to linear and circular forms of pDNA (Figure 5, Lanes 2 and 4). The reactions have been followed over time analyzing the solution, at 0 and 30 min of exposure, by agarose gel electrophoresis. The antioxidant property of ALA and DADS as scavengers of radicals is relevant for the inhibition of DNA damage. However, BAD-NE shows a higher antioxidant property compared to ALA and DADS alone and their mixture, preserving the supercoiled form of pDNA, as shown in Figure 5B. The albumin in the BAD-NE could play a significant role in the antioxidant effect, not only for the possible presence of sulfane sulfur bound to the protein-component of the NE, but also for the tight binding of Cu^2+^ by plasma proteins, including albumin, that effectively blocks the reduction of Cu^2+^ by ascorbate [72, 73] resulting in a relatively low-level of ascorbate radicals production.

Another important antioxidant effect has been observed using the BSA protein of the NEs. The presence of DADS and ALA in the protein mixture also inhibits the protein-protein cross-linking due to the ROS formation by sonication during the NE synthesis. Figure 5C shows the SDS-PAGE of BAD-NE, BD-NE and albumin microbubbles (MBs), obtained both with the same time of sonication and BSA concentration. MBs sample (Figure 5C Lane 4) shows the presence of bands of albumin at high molecular weight that are absent in BD-NE and BAD-NE samples (Figure 5C, Lanes 2 and 3), demonstrating the inhibition of protein cross-links formation in the presence of DADS or ALA and DADS.

The radical scavenging property of BAD-NE has been further investigated by inhibition of free-radical polymerization of PEG-fibrinogen hydrogel induced by addition of Irgacure 2959 photo-initiator and UV (365 nm) exposure [74, 75]. Figure 5D shows the effects of the presence of 8% BAD-NEs v/v in the PEG-fibrinogen mixture with 0.1% w/v of Irgacure 2959.

Altogether these results demonstrate the antioxidant properties of BAD-NE that can be related to both the conserved antioxidant properties of ALA and DADS in the emulsions and to their H_2_S-releasing ability. Several other studies have demonstrated the antioxidants properties of H_2_S-relasing agents and also the ability of this *gasotransmitter* to reduce the oxidative stress through two distinct mechanisms: i) direct scavenging of ROS, increasing the intracellular levels of reduced glutathione (GSH) [76, 78], and ii) up-regulation of endogenous antioxidants through a nuclear-factor-E2-related factor-2 (Nrf2)-dependent signaling pathway [79]. Therefore, the antioxidant effect of BAD-NE on the cells remains to be investigated.

### BAD-NE affects the proliferation of human tumor MCF-7 and HuT 78 cell lines

**Figure 6:**
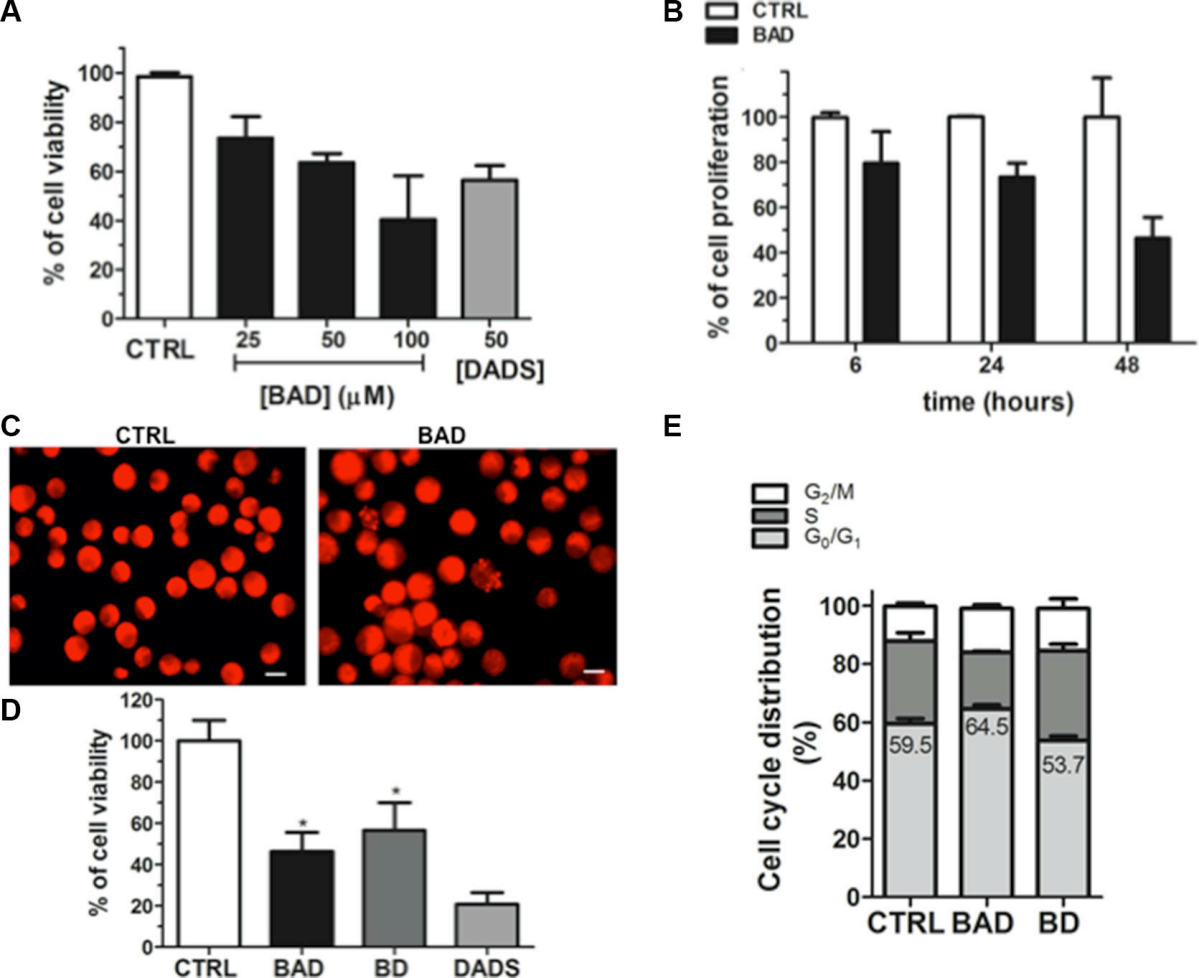
Effects of BAD-NE on cell viability of MCF-7 cancer cell line (**A**) Cell viability of MCF-7 cell line after 24 h of treatment with different concentrations of BAD-NE and 50 μM DADS. The BAD concentrations are expressed as DADS concentration in BAD-NE. (**B**) Cell viability of MCF-7 cell line over the time (at 6 h, 24 h and 48 h) in the presence of BAD-NE with 50 μM of DADS. (**C**) Fluorescence microscope micrographs of MCF-7 cells after 24 h of treatment with BAD-NE (with 50 μM of DADS), the nucleus are stained with PI solution, obtained with mag. 100×, scale bar = 10 μm; (**D**) effects on cell viability of MCF-7 cells after 48 h of treatment with BAD-NE, BD-NE and DADS, at 50 μM of DADS concentration; (**E**) cell cycle distribution of the alive MCF-7 cells after 48 h of treatment with BAD-NE (with 50 μM of DADS) obtained by FACS analyses. The *p* values were < 0.05 with respect to the control using One-way-ANOVA (*n* = 3 or 5 experiments as biological replicas).

**Figure 7:**
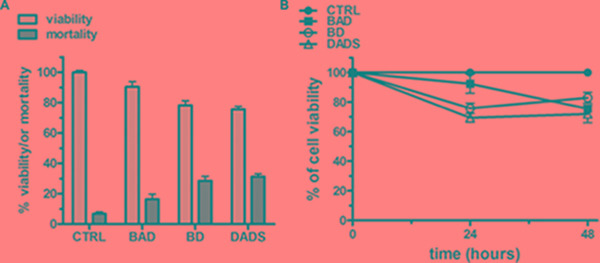
Anti-proliferative effects of the NEs on HuT 78 cancer cell line (**A**) Cell viability and mortality of HuT 78 cell line after 24 h of treatment with BAD-NE, BD-NE and DADS, at 50 μM of DADS concentration. (**B**) Cell viability over the time after 24 and 48 hours of treatment with BAD-NE, BD-NE and DADS (50 μM of DADS). The *p* values were < 0.05 with respect to the control (*n* = 3 or 5 experiments as biological replicas).

**Figure 8:**
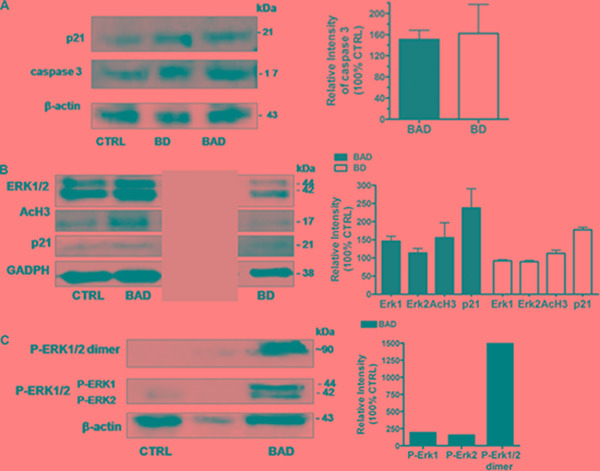
Western blotting analysis of MCF 7 cell line after 24 h of treatment with BAD-NE (**A**) Expression of cleaved form of caspase-3 and p21 protein after BAD-NE (BAD) and BD-NE (BD) treatment; (**B**) Expression of ERK1/2, AcH3, p21 and GAPDH proteins (gray box indicates to unrelated lanes on the same blot, see Figure 3S; (**C**) expression of phosphorylated form of ERK1/2, P-ERK1/2 dimer and β-actin proteins. The relative densitometries of the samples with respect to the control have been obtained after normalization of the concentrations with respect to GAPDH or β-actin concentrations. Each bar represents the ± SD of three experiments as biological replicas.

The inhibition of tumor cell proliferation from OSCs has been also associated with the H_2_S-release through the stability/activity of enzymes involved in the proliferation of neoplastic cells [80, 81]. For instance, the H_2_S slow-releasing donor GYY4137 inhibits tumor growth both *in vitro* and *in vivo* by a combination of cell cycle arrest and cell death suggesting a potential application of H_2_S slow-releasing donor as antitumor agents [82]. The NO- and H_2_S-releasing agent NOSH–aspirin (named NOSH–ASA or NBS-1120), which belongs to the non-steroidal anti-inflammatory drugs (NSAIDs), is effective *in vitro* and in animal models of various cancers [83]. NOSH–aspirin also exhibits significantly reduced adverse gastrointestinal effects and is already in preclinical stage of development as antitumor drug [84]. We have therefore decided to investigate the effects of the BAD-NEs on cell growth using two distinct human tumor cell lines: MCF-7 mammary adenocarcinoma and HuT 78 T-cell lymphoma cells, growing respectively as adherent and suspension cultures. The treatment with BAD-NE (50 μM of DADS and ALA) on both cancer cell lines results into a statistically significant decrease of the cell viability in a concentration and time dependent manner (Figures 6A, 6B and 7A) and MCF-7 cells show more sensitivity to BAD-NE treatment. Although, at same concentration DADS shows a clear anti-proliferative effect, a statistically significant cancer cell death has been observed after BAD-NE treatment. In particular, BAD-NE (50 μM of DADS and ALA) decreases MCF-7 viability by 36.46 ± 5.17% (24 h) and 53.68± 15.16% (48 h) with cell blebbing and chromatin condensation (Figure 6A and 6C). The absence of ALA in the NE leads to a decrease of cell viability by 43.37±16.70% after 48 h of BD-NE (50 μM of DADS) exposure (Figure 6D), which is lower than that observed after BAD-NE treatment. FACS analysis of MCF-7 cells at 48 h treatment with BAD-NE shows the presence of increased sub-G_1_ events with an arrest of the cell cycle in G_0_/G_1_ phase and a decrease in S phase by 8.8% (Figure 6E). Although the increase in sub-G1 phase and the apoptotic blebbing has been also observed after BD-NE treatment, the different effect on the cell cycle observed might suggest that the anti-proliferative effect of BAD-NE is due to the ALA property to trigger cell cycle arrest of the MCF-7 cells at the G_0_/G_1_ phase. This could be in agreement with the described effects of EPA and DHA treatment on cancer cell lines, with down-regulation of cyclin-dependent kinases and cyclins [85]. Interestingly, DADS alone shows a higher cytotoxic effect than both BAD- and BD-NEs (Figure 6D). After both NE treatments, the cell death of the MCF-7 cells is induced, with mitochondria-mediated caspase 3 activation (Figure 8A). These data are in agreement with the observed ability of the garlic derived compounds (DADS, DATS and ajoene) and, more in general, of H_2_S-donors to induce apoptosis in a variety of cancer cells by triggering the mitochondrial-dependent caspase cascade [33, 69, 86–96], with cytosolic release of cytochrome c, disruption of mitochondrial membrane potential and activation of caspase 3 [86, 90–92, 97]. BAD-NE increases the expression of both ERK1 and ERK2, more relevant for ERK1, and of their phosphorylated forms (Figure 8B and 8C). Moreover, Figure 8C shows the presence of a 90 kDa band, visualized by the anti-P-ERK1/2 antibody, suggesting the formation of the P-ERK1/2 dimer after BAD-NE treatment. ERK, belonging to the MAPK super family, is activated in response to a wide variety of growth factors and mitogens and mediates the signal transduction involved in cell proliferation, differentiation and migration [98]. Paradoxically, although ERK pathway is normally associated with enhanced cell proliferation, many studies on bioactive food components have shown that ERK activation up-regulates the expression of cell death genes [99–102]. Furthermore, the activation of ERK in cancer cells by antioxidant chemopreventive compounds (e.g. resveratrol and quercetin) results in anti-proliferative effects with deregulation of apoptosis, senescence or autophagy [103–106]. Activated ERK1/2 translocate to the nucleus where they activate transcription of several genes, including p53 and PUMA, while repressing Bcl-2 [105, 107] and participate in the regulation of G_1_- to S-phase transition [108]. Sustained ERK phosphorylation may act as a compensatory mechanism, mediating the block in G_0_/G_1_ progression in MCF-7 cells treated with BAD-NE. Accordingly, sustained ERK activation can lead to cell cycle arrest at G_0_/G_1_ or G_2_/M [109–115]. More recently, the nuclear translocation of ERK1/2 has been suggested to be an anticancer drug target [116]. In particular, ERK1 might act as negative regulator of cell proliferation, as suggested by Vantaggiato et al., in cell proliferation of fibroblasts by restraining ERK2-dependent signaling [116]. The role played by ERK signaling in the apoptotic response of the cancer cells only after BAD-NE treatment could be related to the combined effect of H_2_S-donor and ALA. Most of the *in vitro* and *in vivo* studies show a reduction in cell/tumor growth from ALA and ALA-rich flaxseed oil treatment [118–121], although this is not confirmed by other reports that show minimal effects [122, 123]. In our studies, the presence of ALA (at 50 μM) in the NEs seems to reduce the anti-proliferative effect in cancer cells when compared to BD-NEs treatment after 24 h (Figure 7B), but it seems to increase after 48 h (Figures 6D and 7B). This effect could be also related to the sustained ERK1/2 phosphorylation. Thus, the presence of ALA increases the effect on this signaling pathway, resulting in a significant change of the mechanism of the cell growth arrest respect to DADS.

Our results are in agreement with recent studies where ALA treatment (75 μM, 72 h) reduces the cell growth of MCF-7 cells [124]. The authors suggests that ALA affects cell cycle, grow factors signaling, oxidative stress, anti-estrogenic activity or RNA/micro RNA expression [124]. Additionally, ALA may be incorporated in lipid rafts of the cell membrane, affecting cell signaling and growth [124–126].

Along this line, we observed significant increases of p21 and acetylated histone H3 (AcH3) expression (Figure 8A and 8B) after BAD-NE treatment. The accumulation of p21 may be a consequence of the ERK1/2 activation. The magnitude of the ERK1/2 signal, in fact, can play a key role in determining the final effect in cell proliferation [127, 128]. Indeed, a strong activation of ERK1/2 by active Ras or Raf leads to cell cycle arrest in cell line models by inducing the expression of the Cdk inhibitor p21. Therefore, the persistent up-regulation of p21, with inhibition of Cdk4 and Cdk2 activity, results in G_1_ arrest [129–133].

Our results also suggest a modulation of gene expression by histone hyperacetylation in response to both BAD-NE and BD-NE treatments (Figure 8B). This effect might be related to the presence of DADS and its effect on histone acetylation, in agreement with previously works on hyperacetylation by DADS [134–136].

### BAD-NE stability over time

**Figure 9:**
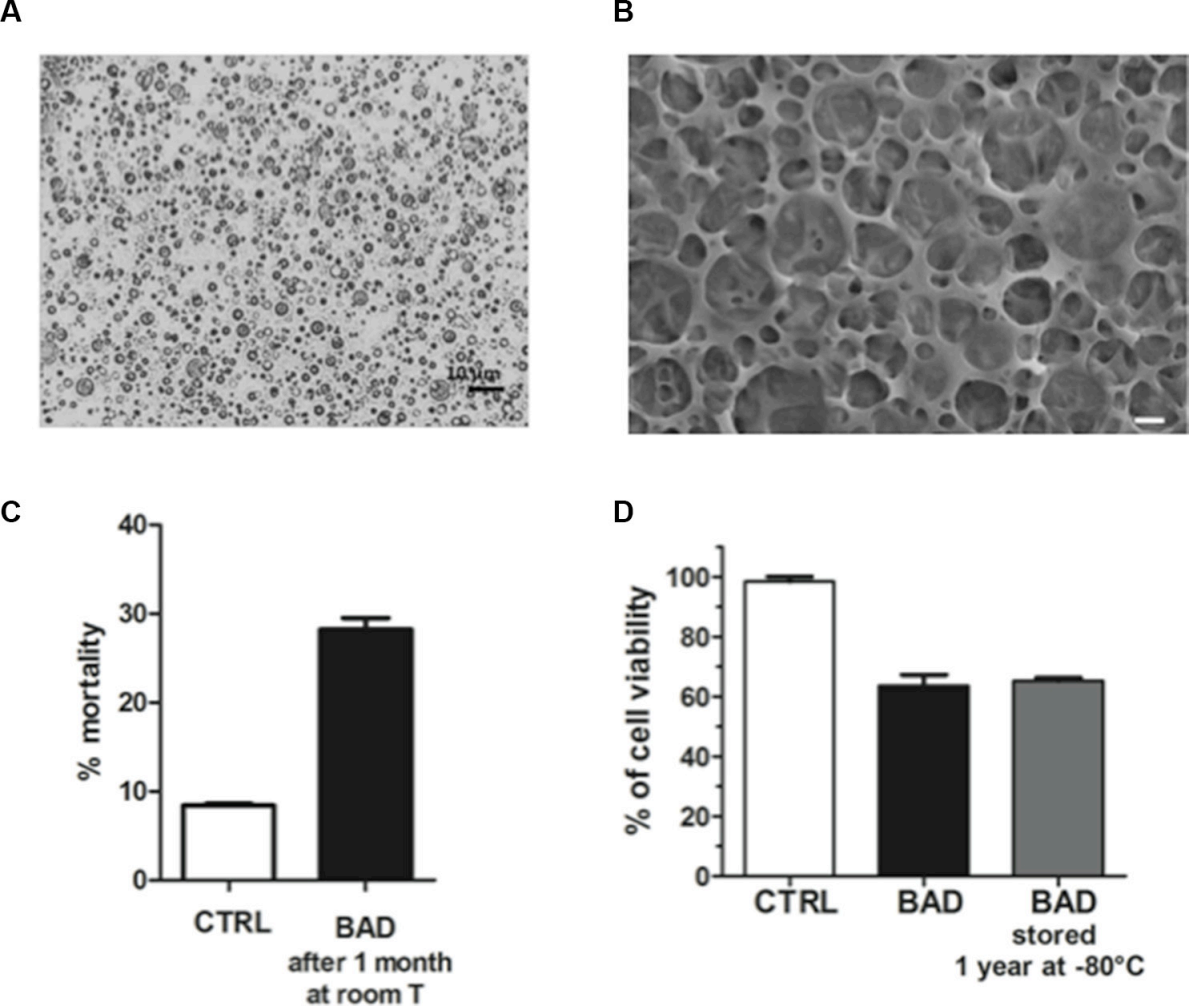
Stability of the BAD-NEs over time (**A**) Optical micrograph of BAD-NE after one month at room temperature, obtained at mag. 100×, scale bar =10 μm; (**B**) representative HR-SEM micrograph of BAD-NEs kept for 1 month at room temperature, obtained at 10 kV, scale bar = 1 μm; (**C**) cell mortality of MCF-7 after 48 h of treatment with BAD-NEs kept for 1 month at room temperature (about 25°C) (the estimated concentration of DADS was 15 μM). (**D**) Cell viability of MCF-7 cells after 48 h of treatment with fresh preparation of BAD-NE and stored one year at –80°C (with 50 μM of DADS).

The stability over time of BAD-NE has been assessed by optical and SEM microscopy as well as by proliferation assay. Figure 9A and 9B show the optical and SEM micrographs of BAD-NE kept on the bench for 1 month at room temperature. The effect on proliferation has been maintained, as shown in Figure 9C; only 14% of the anti-proliferative activity is lost, compared to fresh NEs. Moreover, the storage at –80°C for one year does not affect any activity on cancer cells (Figure 9D). The stability over time at room temperature and the easy storage of BAD-NE make this new formulation a good model for producing drugs.

### Effects of BAD-NEs on growth of human Lin^–^ Sca-1^+^ cardiac progenitor cells

**Figure 10:**
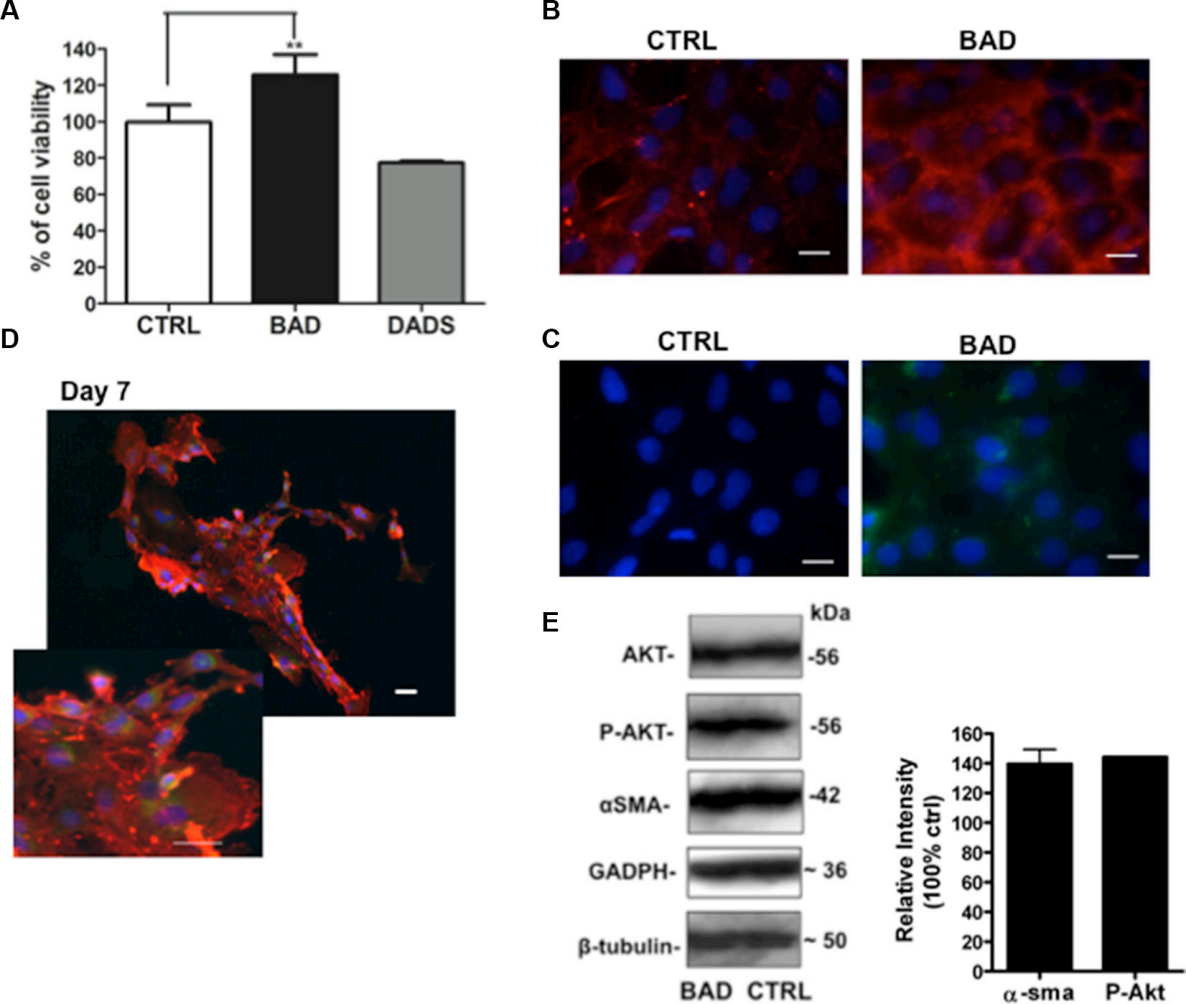
Effects of BAD-NE on cell viability of Lin^–^ Sca-1^+^ hCPC line (**A**) Cell viability of hCPC after 3 days of growth in the presence of BAD-NE (BAD) and DADS (50 μM of DADS). The *p value* < 0.05; *n* = 7 biological replicas; (**B**) fluorescence micrographs of hCPCs cultured for 3 days in the presence of BAD-NE. The nuclei are stained with Hoeschst 33342 (in blue) and the expressions of α-sma (in red) and Cx43 (in green) proteins are detected. Scale bars = 20 μm; (**E**) western blotting analysis of Lin^-^ Sca-1^+^ hCPC line after 3 days in the presence of BAD-NE, the expression of Akt, P-Akt , α-sma and GAPDH and β-tubulin proteins are assessed. The relative densitometries of P-Akt, α-sma of the treated with respect to the control have been obtained after normalization of the concentrations with respect to β-tubulin concentration.

Beneficial effects of omega-3 fatty acids and garlic OSCs on the cardiovascular system have been reported by numerous clinical studies. Moreover, several reports indicate that H_2_S-donors stimulate cell proliferation in rat cardiac myocytes and interstitial cells of Cajal [137, 138]. For these reasons, we set up to assess the effects on cell proliferation and on the expression of proteins involved in cell differentiation. To this end, we investigated the effect of BAD-NE on Lin^-^ Sca-1^+^ hCPCs. The cell viability of hCPCs has been monitored after 3 and 6 days of incubation in cell culture medium containing BAD-NE. A significant increase in cell viability has been observed (Figure 10); in particular, after only 3 days of treatment with BAD-NE (50 μM DADS) a significant increase of the cell viability up to 125.72 ± 11.12% (*p <* 0.05; *n* = 7 biological replicates) (Figure 10A) is detected. Conversely, DADS treatment decreases the cell viability by 77.37 ± 0.91% (Figure 10A). Furthermore, BAD-NE treatment of hCPCs up-regulates α-smooth muscle actin (α-sma) (Figure 10B, 10D and 10E) and connexin-43 (Cx43) proteins (Figure 10C). Cx43 is an essential protein in the formation of hemichannels and gap junctions, facilitating electrical coupling between cells in the myocardium [139, 140]. Although, Cx43 expression may increase progressively in the cardiac microenvironment, it is extremely low in mesenchymal stem cells (MSCs) [141]. Previously, studies have shown that high Cx43 expression in MSCs improves cell survival, cardiomyogenesis and heart function following transplantation [142, 143]. Cx43 overexpression promotes survival of MSCs and cardiac function preservation in the ischemic heart [143]. Previous studies, including a clinical trial, demonstrated that Cx43 may be used to reduce proarrhythmogeny of myoblast transplantation [144–147]. Moreover, an improvement of preservation of ischemic hearts was shown using MSCs overexpressing Cx43 [142, 148]. Altogether these studies suggest that overexpression of Cx43 may contribute to the therapeutic efficacy of cardiac progenitor/stem cells transplantation. Moreover, the transplantation of MSCs overexpressing Cx43 results in reduced infarct size and greater functional improvement [142, 148]. Notably, MSCs overexpressing Cx43 promote neovascularization, probably through secreting more angiogenic cytokines, improve heart function and reduce infarct size [143]. Thus, due to the increase of Cx43 expression, BAD-NE treatment may lead to positive effects on resident stem cells and also to a useful pretreatment for improving the transplantation of MSCs in tissue repair. Finally, BAD-NE treatment induces the increase of phosphorylated form of Akt (Figure 10E); this is in keeping with the reported increase of the endothelial cell growth, migration, wound healing, capillary morphogenesis by Akt signaling pathway promoted by H_2_S-releasing agents [149, 150]. The effects, here described, seem related to both the H_2_S release and ALA. The treatment with 50 μM ALA of the hCPC, in fact, leads also to an increase of P-AKT, as shown in the Supplementary Figure S4. If H_2_S, in a cell specific manner, increases cell survival signaling and decrease cell death effects [151], ALA seems to exert cardioprotection including anti-inflammatory and anti-oxidative stress effects via activation of Akt [152]. Indeed, in previous studies, using isolated cell models and cardiac tissue of cardio-myopathic hamsters, the intrinsic apoptotic cascade was prevented by ALA treatment by activation of the survival Akt pathway and caveolin 3-modulation [55].

## CONCLUSIONS

**Figure 11:**
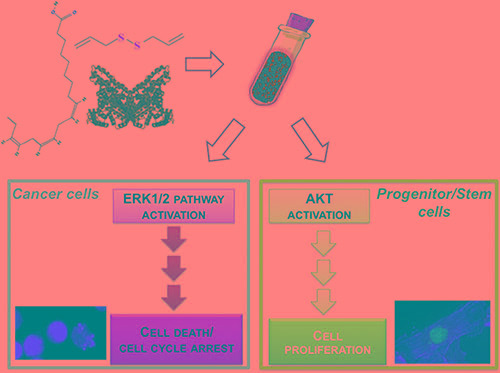
Schematic illustration of BAD-NE preparation and its effects on cancer and adult progenitor stem cells

Here we have produced and characterized a novel nanoemulsion of two natural compounds with relevant effects on both tumor and cardiovascular system. The antioxidant properties and the stability over time of BAD-NE, together with its ability to release H_2_S, confer important characteristics for medical applications to this novel formulation. Our data on two cancer cell lines show a relevant ability to induce apoptosis with mitochondrial depolarization and caspase 3- activation as well as cell cycle arrest at G_0_/G_1_. This effect of BAD-NE is at least in part mediated by the activation of ERK1/2 pathway and up-regulation of p21. This anti-proliferative effect on cancer cell has been not observed in adult progenitor stem cells that, on the contrary, proliferate upon BAD-NE treatment (Figure 11). The cell type selectivity, characteristic of both OSCs and omega-3 fatty acids, is fully preserved in our formulation. In particular the H_2_S-release together with ALA could also stimulate *in vivo* the Akt phosphorylation, improve the survival of stem and normal cells, inhibit the inflammatory and ischemic processes promoting the tissue repair and the onset of neoplasia.

## MATERIALS AND METHODS

### NEs preparation

NEs were prepared using a 5% w/v of Bovine Serum Albumin (BSA) water solution with 0.1% of β-mercapto-ethanol (Sigma-Aldrich, Italy), 100 μl/ml DADS (Sigma-Aldrich, Italy) and with or without 100 μl/ml of ALA (Sigma-Aldrich, Italy) for BAD-NE and BD-NE synthesis, respectively. The mixtures were treated with 20 kHz ultrasounds applied for 60s at the air-water interface, using a Sonics and Materials ultrasound generator (Branson) with a 3 mm in diameter horn at an applied acoustic power of 160 Wcm^–2^. After sonication, 12 ml of ddH_2_O were added to the samples and then incubated at 4°C for about 48 h. NEs were separated from the remaining protein and broken microparticles by flotation and repeated washing. The milky suspensions containing BAD-NEs (at the top) or BD-NE (at the bottom) were recovered, transferred in a new tube, characterized and stored at –80°C.

### RP-HPLC analyses

RP-HPLC analyses of the NEs and of the single compounds were performed on a LC-10AVP (Shimadzu, Milan, Italy) with a solvent B gradient (0–5 min, 0%; 5–25 min, 60%; 25–55 min, 90% and 55–75 min 90%), using 0.1% v/v trifluoracetic acid (TFA) as solvent A and 80% v/v CH_3_CN, 0.1% v/v TFA as solvent B, and a C_18_ column (CPS Analitica, 150 × 4.6 mm, 5 μm). Elute was monitored at 220 nm by UV detector (Shimadzu, Milan, Italy).

### ^1^H-NMR spectroscopy

NMR experiments were carried out on a Bruker AVANCE spectrometer (Karlruhe, Germany) operating at 16.4 T. Chemical shifts were referenced internally to tetramethylsilane (TMS, 0 ppm). For ^1^H NMR spectra of samples in CDCl_3_/CD_3_OD, 32K complex data points were acquired, and 128 free induction decay signals (FIDs) were averaged with a 60° observe pulse, preceded by a 5.0 s pre-saturation pulse, for residual HDO signal suppression. The FIDs were zero-filled to 64 K complex data points and the spectra were baseline corrected (in the frequency domain) for peak area integration.

### Optical and fluorescence microscopy analysis of NEs

Microscopy analysis and the size distribution of NEs were performed using a Motic microscope mod. BA310 Digital using magnification 100X.

FITC-BSA solution was obtained using an optimized FITC conjugation protocol [153, 154]. 1 μl of 10 mg/ml FITC in DMSO solution was added to 100 μl of NEs (with 3 mg/ml BSA) in 15 mM sodium bicarbonate, pH 8.0, buffer and the mixture incubated at room temperature for 2 h in dark condition. Using this protocol the coupling efficiency of the fluorochrome to BSA after 2 h of incubation was about 52%, with a final ratio of 2.9/1 fluorochorme/protein moles, calculated as previously described [154, 155]. Protein concentrations were measured with the BCA protein assay (Sigma-Aldrich, Milan, Italy). After labeling the NEs were dialyzed 24 h against ddH_2_O at room temperature (23°C) using a dialysis membrane (18 kDa *cut-off*) (Sigma-Aldrich, Milan, Italy). Finally, the samples of emulsions were mounted on slides and analyzed by fluorescence microscopy. All fluorescence micrographs were performed using Nikon Filter microscope and Lucia G version 4.61 software.

### HR-SEM analysis

NEs were characterized using a Zeiss Leo Supra 35 field emission gun scanning electron microscope (FEG-SEM, Cambridge Leo Supra 35, Carl Zeiss). The samples were placed on carbon tape, dried and metalized under vacuum before SEM. Specific imaging detectors were chosen, accordingly.

### H_2_S assay

The H_2_S production by NEs was assessed using methylene blue assay [20, 155, 156]. The total volume of the reaction solution was 150 μl and it was constituted of 3 mM sodium thiosulfate, 1 mM DTT and 50 mM Tris HCl, pH 7.4 buffer. The mixtures were incubated at 37°C for 30 min, while shaking on a rotary shaker to facilitate the release of H_2_S gas from the aqueous phase. The reaction was activated by the addition of 20 μl of solution I (20 mM N′,N′-dimethyl-p-phenylene-diamine-dihydro-chloride in 7.2 M HCl) and 20 μl of solution II (30 mM FeCl_3_ in 1.2 M HCl). After 10 min incubation at room temperature, coupled to a gentle mixing, the absorbance of the solution was detected at 670 nm. The standard curve for H_2_S was prepared using 0–350 μM Na_2_S as H_2_S source (Supplementary Figure S2). The following steps were the same as described above. The results were plotted using GraphPad Prism version 5.0 for Windows (GraphPad Software, San Diego, CA, USA).

### pDNA cleavage inhibition

Plasmid DNA (pQE30-GSTWW) was cloned and isolated from *E.coli* BL21 strain using Plasmid Mini Kit (Sigma-Aldrich, Milan, Italy). The plasmid DNA cleavage reactions were performed in a total reaction volume of 10 μl containing cleavage agent and 0.5 μg of pDNA for each sample. Final concentrations were 10 mM ascorbic acid and 100 μM CuCl_2_ in the presence and in the absence of 1 μl of BAD-NEs (30 mM ALA and 32 mM DADS) in 50 mM Tris HCl, pH 7.5, sterile buffer. After equilibration for 5 minutes at room temperature, the reactions were initiated by the addition of the ascorbic acid. The reactions were carried out at 37°C and were terminated by addition of 2 μl of 6× gel loading buffer (5% glycerol, 0.125% bromophenol blue, 25 mM EDTA) and placed on ice before electrophoresis in 1% agarose gel in the TAE buffer. Samples were run for 120 min at 70 V and stained with ethidium bromide.

### Analysis of anti-oxidant properties

The effects of the ultrasounds on the 5% w/v BSA protein solutions in the presence and in the absence of DADS and ALA were assessed by SDS-PAGE in 15% polyacrylamide gel of BD-NE, BAD-NE and MBs emulsions.

The cross-linking of the PEG-fibrinogen (8 mg/ml) into hydrogel was achieved as described by Almany et al [74, 75]. The free-radical polymerization was obtained by addition of 0.05 or 0.1% w/v of a photo-initiator Irgacure^®^ 2959 (Ciba Specialty Chemicals, Basel, Switzerland) using a stock solution containing 10% w/v Irgacure^®^ 2959 in 70% v/v ethanol, and exposing the sample to long-wave UV light (365 nm, 5 mW/cm^2^) for 5 min. The material undergoes a phase change from liquid to gel. The photo-polymerization was performed in the absence and in the presence of 8% v/v BAD-NE (32 mM ALA and 30 mM DADS).

### Cancer cells proliferation

The human breast adenocarcinoma MCF-7 cells (0.1 × 10^5^/cm^2^) and human T-cell lymphoma HuT 78 cells (0.2 × 10^5^/ml) (gift of Dr. Iorio at Istituto Superiore di Sanità, Rome, Italy) were pre-incubated for 24 hours in RPMI medium 1640 (GIBCO, Italy) in the presence of 1% glutamine, 10% heat-inactivated fetal bovine serum (FBS) (Sigma-Aldrich, Italy) and antibiotics (1% penicillin and streptomycin sulfate) at 37°C in air supplemented with 5% CO_2_. MCF-7 cells were sub-cultured by enzymatic digestion with 0.25% trypsin/1 mM EDTA solution when they reached approximately 60–80% confluence. MCF-7 and HuT-78 cells in logarithmic growth phase were treated with different concentrations of BAD-NE (0–100 μM) or 50 μM of BD-NE. Cells were collected each day after 6, 24 and 48 h, and counted under a light microscope after trypan blue staining (0.4% Tripan blu solution, Sigma-Aldrich, Milan, Italy) using a Thoma chamber. The rates of growth inhibition were calculated with respect to the control culture taken as 100% growth. There were at least two biological replicates for each concentration and the experiment was performed three times. Rates of growth inhibition with respect to the control culture taken as 100% growth were calculated and the percent of cell viability was also performing by MTT assay [157]. For the nuclei analysis by microscopy the cells after the treatment were fixed with 4% paraformaldehyde for 20 minutes followed by incubation for 15 minutes with propidium iodide (Sigma-Aldrich, Italy) solution and washing with PBS buffer. The cells were mounted on slides and analyzed using fluorescence microscope (Nikon, Filter) and Lucia G version 4.61 software.

### Cell cycle analysis

The cell cycle distribution was measured by flow-cytometry. The harvested cells (about 0.5 × 10^6^ cells) were stained with 50 μg/ml propidium iodide (Sigma-Aldrich, Milan, Italy) in PBS buffer with 0.1% Triton X-100 and 1 mg/ml sodium citrate. Then, they were immediately analyzed using a flow cytometer FACSCalibur (Beckton and Dickinson, San Josè, CA, USA) and the percentage of cells in each phase of cell cycle was evaluated according to Nicoletti et. al [158].

### Protein extraction and Western blot analysis

Proteins were extracted from MCF-7 cells in 100 μl of RIPA buffer containing a protease inhibitors' cocktail (Sigma-Aldrich) and pervanadate as phosphatase inhibitor and sonicated for 10 sec incubating in ice. Samples were centrifuged for 10 minutes at 8000 rpm at 4°C. Protein contents were determined by BCA protein assay (Sigma-Aldrich, Milan, Italy), cell extracts (30 μg of protein) were electrophoresed on 12 or 15% polyacrylamide gel, electro-blotted on PVDF membrane (Applied Biosystem, Milan, Italy). The membrane was then blocked and probed with primary monoclonal antibodies (Ab-β-actin mouse, Ab-GAPDH rabbit, Ab-AcH3 rabbit; Ab-ERK1/2 rabbit, Ab-P-ERK1/2 rabbit, Ab-p21^CIP1/WAF1^, Ab-caspase-3 rabbit 17 kDa fragment, Ab-p38 mouse, Ab-P-p38 mouse, Ab-Akt and Ab-pAKT-(pSer426) rabbit, Ab-β-tubulin mouse, Ab-Cx43 mouse, Ab-a-sma mouse) (Sigma-Aldrich, Italy) overnight at 4°C. Immunoblots were probed with secondary antibodies (Sigma-Aldrich, Italy) for 2 hours at room temperature. Immunoblots were probed with Ab-β-actin, Ab-GAPDH or Ab-β-tubulin (Sigma-Aldrich Italia, Milan Italy) for controlling the protein loading. The protein complexes were formed upon incubation with specific secondary antibodies (dilution 1:10000) (Sigma-Aldrich, Milan, Italy). Western blots were probed with a Super Signal West Pico kit (Thermo Scientific, USA) to visualize signal, followed by exposure to X-ray film (Kodak, Sigma-Aldrich, Italy) or using a Fluorchem Imaging system (Alpha Innotech Corporation-Analitica De Mori, Milan, Italy).

### Human cardiac progenitor cells proliferation

Cell studies were conducted on human Lin^-^ Sca-1^+^ cardiac progenitor cells (hCPC) [159, 160], which were isolated from auricular biopsies made during the course of coronary artery bypass surgery from patients after signing a written consent form. Cell cultures were performed in Dulbecco's Modified Eagle Medium (DMEM) (Gibco, Italy), containing 10% v/v Fetal Bovine Serum (FBS) (Gibco, Italy), 1% w/v penicillin-streptomycin (Sigma-Aldrich, Italy), 1% w/v L-Glutamine (Gibco, Italy) and 1% v/v non-essential amino acids solution (Sigma-Aldrich, Italy). To assess the hCPC phenotype after the treatment the cells were washed in PBS, fixed in 4% v/v PFA in PBS for 15 min at 4°C, permeabilized with 0.2% v/v Triton X-100 (Sigma-Aldrich, Italy) for 10 min and incubated with antibodies for connexin-43 (Cx43) mouse and α-smooth muscle actin (α-sma) mouse (Sigma-Aldrich, Italy), followed by the appropriate 488-Alexa fluorochrome-conjugated secondary antibodies (Invitrogen, Italy). Nuclei were stained with Hoechst 33342 (Sigma-Aldrich, Italy). The cells were mounted on slides and analyzed by fluorescence microscopy using a Nikon Filter microscope and Lucia G version 4.61 software.

### Statistical analysis

The statistical analysis was performed using GraphPad Prism version 5.0 for Windows (GraphPad Software, San Diego, CA, USA). Data from three, five or seven independent experiments were quantified and analyzed for each variable using a one-tailed Student's *t-test* or ANOVA one-way test. A *p value* of < 0.05 was considered to be statistically significant. Standard errors of the mean were calculated and presented for each type of sample.

## SUPPLEMENTARY MATERIALS FIGURES AND TABLES



## Abbreviations

3-MTS: 3-mercapto-piruvate sulfurtrasferase
α-sma: α-smooth muscle actin
A375 cells: melanoma cell line
AcH3: acetylated histone H3
ALA: alpha-linolenic acid
Akt: Protein Kinase B
BAD-NE: BSA/ALA/DADS nanoemulsion
BCA: bicinchoninic acid
BD-NE: BSA/DADS nanoemulsion
BSA: Bovine serum albumin
CBS: cystathionine β-synthase
CSE: cystathionine γ–lyase
Cx43: connexin-43
DADS: diallyl-disulfide
DMEM: Dulbecco's Modified Eagle Medium
DATS: diallyl-trisulfide
DU145 cells: prostate cancer cell line
ERK1/2: extracellular signal–regulated kinases 1/2
ESCC cells: oesophageal squamous cancer cell line
FBS: Fetal Bovine Serum
FID: free induction decay signal
FITC: fluorescein isothiocyanate
H2S: hydrogen sulfide
hCPC: human Lin-Sca-1+ cardiac progenitor cells
H358: human lung cancer cell line
H460 cells: human lung cancer cell line
HuT 78: human T-cell lymphoma cell
HR-SEM: high-resolution scanning electron microscopy
MBs: albumin microbubbles
MCF-7: breast cancer
MDA-MB-231 cells: breast cancer cell line
MSCs: mesenchymal stem cells
NE: nanoemulsion
NSAIDs: non-steroidal anti-inflammatory drugs
OSCs: Organo-Sulfur Compounds
p21: cyclin-dependent kinase inhibitor 1
p38: p38 mitogen-activated protein kinases
PC-3 cells: prostate cancer cell lines
pDNA: plasmid DNA
PUFA: poly-unsaturated fatty acids
SEM: scan electro-microscopy
TFA: trifluoroacetic acid
TST: thiosulfate: cyanide sulfurtransferase enzyme.
