# Supplementary material for: H2S-releasing nanoemulsions: a new formulation to inhibit tumor cells proliferation and improve tissue repair
Source: Oncotarget. 2016 Oct 12;7(51):84338–58. doi: 10.18632/oncotarget.12609 (PMC5356665; doi:10.18632/oncotarget.12609)
Supplement: Supplementary file 1 [file oncotarget-07-84338-s001.pdf]

H_2S-releasing nanoemulsions: a new formulation to inhibit tumor cells proliferation and improve tissue repair

Supplementary Materials

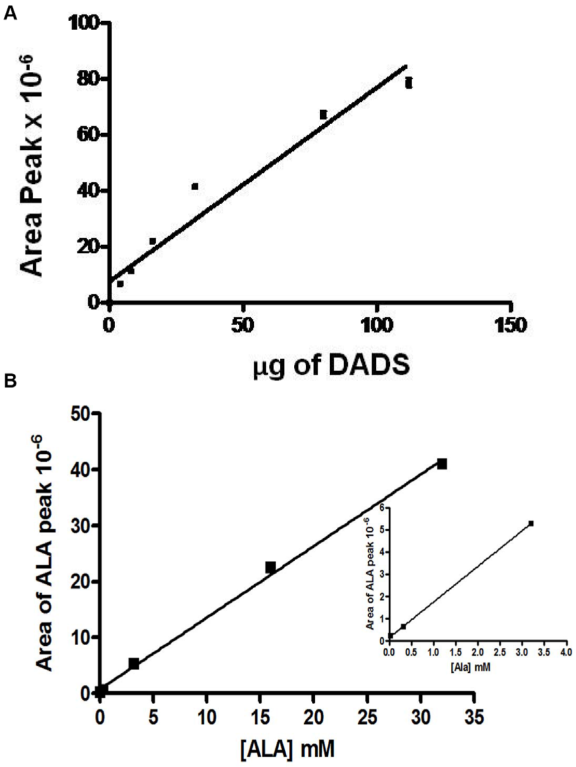

Supplementary Figure S1: Calibration curves of DADS and ALA by RP-HPLC. (**A**) Area of the characteristic peak of DADS is reported as function of the DADS concentration of the sample; (**B**) area of the characteristic peak of ALA is reported as function of the ALA concentration of the sample.

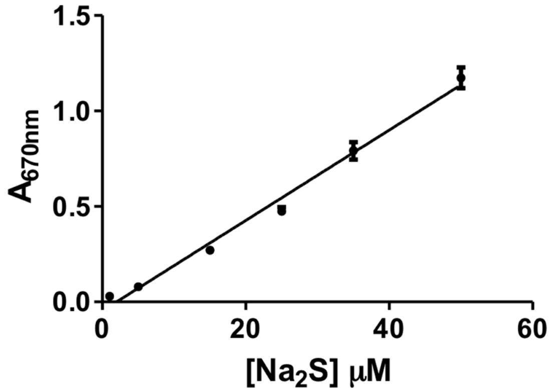

Supplementary Figure S2: Calibration curves of H_2S. Calibration curve of H_2S formation by methylene blue assay, obtained at different concentration of Na_2S. Absorbance 670 nm. Each bar represents the ± SD of three experiments.

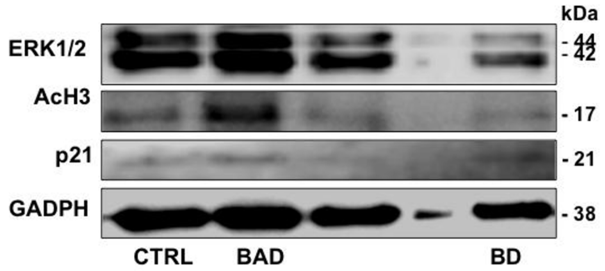

Supplementary Figure S3: Figure 5b without the gray box. Western blotting analysis of MCF 7 cell line after 24 h of treatment with BAD-NE, expression of ERK1/2, AcH3, p21 and GAPDH proteins.

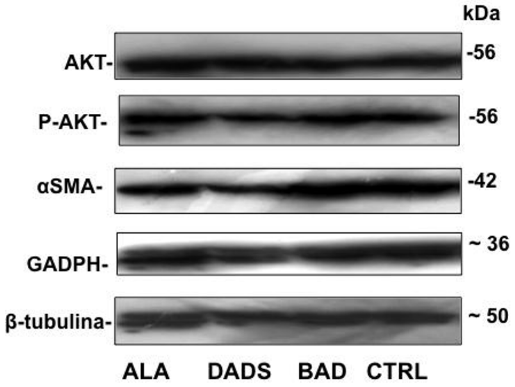

Supplementary Figure S4: Full blot, as shown in Figure 10E, including the protein expression after ALA and DADS treatments. Western blot analysis of Lin$^-$ Sca-1$^+$ hCPC line after 3 days in the presence of 50 µM of ALA, 50 µM of DADS, 50 µM of BAD-NE (BAD) and control the expression of Akt, P-Akt , α-sma and GAPDH and β-tubulin proteins.
